# Supplementary material for: Endothelin-3 and T-type Ca2+ channels drive enteric neural crest cell calcium activity, contractility and migration
Source: Nat Commun. 2026 Jan 20;17:1370. doi: 10.1038/s41467-025-68121-5 (PMC12876838; doi:10.1038/s41467-025-68121-5)
Supplement: Supplementary file 2 — Description of Additional Supplementary Files [file 41467_2025_68121_MOESM2_ESM.pdf]

Description of Additional Supplementary Files

File Name: Supplementary Movie 1
Description: CA across stages E10.5, E11.5, E12.5 at the migration front

File Name: Supplementary Movie 2
Description: Registration of CA with Sox10 (ENCCs) and Tuj1 (neurons), E11.5 ileum

File Name: Supplementary Movie 3
Description: Effect of EDN3 1 nM & 10 nM in E11.5 ilcc and effect of EDN3 10 nM in E12.5 ileum

File Name: Supplementary Movie 4
Description: Effect of EDNRB blocker BQ788 at E11.5 on CA, and long-term morphological effects

File Name: Supplementary Movie 5
Description: Effect of extracellular Ca^{2+} removal by EDTA 2 mM, and subsequent stimulation by EDN3

File Name: Supplementary Movie 6
Description: Effects of T-type Ca^{2+} channel blocker Z944, CaV3.2 specific inhibitor ascorbic acid and CaV3.1 & CaV3.3 agonist SAK3.

File Name: Supplementary Movie 7
Description: Effects of Cl^- channel blocker NFA and NPPB.

File Name: Supplementary Movie 8
Description: Network-spanning rise in intracellular Ca^{2+} induced by ATP_e 1 mM

File Name: Supplementary Movie 9
Description: Mesenchyme backflow during ENCC invasion of the colon, E11.5 followed for 24 h. The video needs to be loaded in ImageJ and the time-cursor tracked fast-forward & backward repeatedly between frames 70 and 89, focusing on the areas indicated by the arrows.

File Name: Supplementary Movie 10
Description: Lamellipodium detachment after Ca^{2+} transient

File Name: Supplementary Movie 11
Description: Deep learning assisted tracking of cell nucleus boundary and centroid of an ENCC chain with calcium activity.

File Name: Supplementary Movie 12
Description: ENCC 3D traction force on collagen gel is stimulated by EDN3 and relaxed by BQ788

File Name: Supplementary Code 1
Description: ImageJ macro that lays a fixed grid over a time-lapse video of Ca2+ transients, and outputs intensity as a function of time in every box of the grid, computes the area occupied by GCaMP positive cells in the field of view by iterative Bernsen thresholding and

outputs the resulting masks that can be checked individually. The output is designed to be fed in the Matlab script "MatlabProgram_Batch_Analysis_of_ImageJMacroOutput" for final data output.

File Name: Supplementary Code 2
Description: Matlab script that outputs heatmaps & average Ca2+ event number, frequency, width, intensity ratio. To be used downtream of the ImageJ Macro "Batch_calcium_analysis"
