# Supplementary material for: Endothelin-3 and T-type Ca2+ channels drive enteric neural crest cell calcium activity, contractility and migration
Source: Nat Commun. 2026 Jan 20;17:1370. doi: 10.1038/s41467-025-68121-5 (PMC12876838; doi:10.1038/s41467-025-68121-5)
Supplement: Supplementary file 17 — Reporting Summary [file 41467_2025_68121_MOESM17_ESM.pdf]

nature portfolio

Corresponding author(s): Nicolas R. Chevalier

Last updated by author(s): Oct 23, 2025

Reporting Summary

Nature Portfolio wishes to improve the reproducibility of the work that we publish. This form provides structure for consistency and transparency in reporting. For further information on Nature Portfolio policies, see our Editorial Policies and the Editorial Policy Checklist.

Statistics

For all statistical analyses, confirm that the following items are present in the figure legend, table legend, main text, or Methods section.

n/a	Confirmed	
☐	☒	The exact sample size (*n*) for each experimental group/condition, given as a discrete number and unit of measurement
☐	☒	A statement on whether measurements were taken from distinct samples or whether the same sample was measured repeatedly
☐	☒	The statistical test(s) used AND whether they are one- or two-sided<br>*Only common tests should be described solely by name; describe more complex techniques in the Methods section.*
☒	☐	A description of all covariates tested
☒	☐	A description of any assumptions or corrections, such as tests of normality and adjustment for multiple comparisons
☐	☒	A full description of the statistical parameters including central tendency (e.g. means) or other basic estimates (e.g. regression coefficient) AND variation (e.g. standard deviation) or associated estimates of uncertainty (e.g. confidence intervals)
☐	☒	For null hypothesis testing, the test statistic (e.g. *F*, *t*, *r*) with confidence intervals, effect sizes, degrees of freedom and *P* value noted<br>*Give P values as exact values whenever suitable.*
☒	☐	For Bayesian analysis, information on the choice of priors and Markov chain Monte Carlo settings
☒	☐	For hierarchical and complex designs, identification of the appropriate level for tests and full reporting of outcomes
☒	☐	Estimates of effect sizes (e.g. Cohen's *d*, Pearson's *r*), indicating how they were calculated

Our web collection on statistics for biologists contains articles on many of the points above.

Software and code

Policy information about availability of computer code

Data collection	Andor iQ3 (microscope software)
Data analysis	ImageJ (integrated and custom macros & plugins), Matlab, Segment Anything Model 2

For manuscripts utilizing custom algorithms or software that are central to the research but not yet described in published literature, software must be made available to editors and reviewers. We strongly encourage code deposition in a community repository (e.g. GitHub). See the Nature Portfolio guidelines for submitting code & software for further information.

Data

Policy information about availability of data

All manuscripts must include a data availability statement. This statement should provide the following information, where applicable:

- Accession codes, unique identifiers, or web links for publicly available datasets
- A description of any restrictions on data availability
- For clinical datasets or third party data, please ensure that the statement adheres to our policy

Essential data generated or analyzed during this study are included in the manuscript and supporting files. Source data as well as essential codes for calcium imaging analysis are provided with this paper. Other data are available from the corresponding author upon request.

Research involving human participants, their data, or biological material

Policy information about studies with human participants or human data. See also policy information about sex, gender (identity/presentation), and sexual orientation and race, ethnicity and racism.

Reporting on sex and gender	No sex/gender bias (embryo sex was not determined / considered).
Reporting on race, ethnicity, or other socially relevant groupings	N/A
Population characteristics	N/A
Recruitment	N/A
Ethics oversight	Killing of mice for retrieval of embryos is a terminal procedure for which neither CNRS or INSERM assign ethics approval codes hence none are given here.

Note that full information on the approval of the study protocol must also be provided in the manuscript.

Field-specific reporting

Please select the one below that is the best fit for your research. If you are not sure, read the appropriate sections before making your selection.

☒ Life sciences ☐ Behavioural & social sciences ☐ Ecological, evolutionary & environmental sciences

For a reference copy of the document with all sections, see nature.com/documents/nr-reporting-summary-flat.pdf

Life sciences study design

All studies must disclose on these points even when the disclosure is negative.

Sample size	The sample size range is n=5-23, depending on the type and variability of experiments.
Data exclusions	No data were excluded.
Replication	All sample numbers indicated in this report correspond to different embryos (guts, biological replicates). Except for ls/ls experiments which were performed on 2 litters, data for all other experiments was collected from at least 3 different litters and technically replicated at least 3 times, i.e. they were performed on 3 different days with fresh samples following the same procedure each time.
Randomization	N/A
Blinding	N/A

Reporting for specific materials, systems and methods

We require information from authors about some types of materials, experimental systems and methods used in many studies. Here, indicate whether each material, system or method listed is relevant to your study. If you are not sure if a list item applies to your research, read the appropriate section before selecting a response.

Materials & experimental systems

n/a	Involved in the study	
☐	☒	Antibodies
☒	☐	Eukaryotic cell lines
☒	☐	Palaeontology and archaeology
☐	☒	Animals and other organisms
☒	☐	Clinical data
☒	☐	Dual use research of concern
☒	☐	Plants

Methods

n/a	Involved in the study	
☒	☐	ChIP-seq
☒	☐	Flow cytometry
☒	☐	MRI-based neuroimaging

Antibodies

Antibodies used	Anti-SOX10 (Sigma, AMAB91297); βIII-tubulin FITC conjugated antibody (Abcam 224978); α-SMA Cy3 conjugated antibody (Sigma

Antibodies used	C6198); rabbit anti CaV3.1 (Alomone Labs #ACC-021) ; 1:100 rabbit anti CaV3.2 (Alomone Labs #ACC-025); 1:100 rabbit anti CaV3.3 (Alomone Labs #ACC-009)
Validation	From IHC appearance (signal on ENCCs versus signal in mesenchyme)

Animals and other research organisms

Policy information about studies involving animals; ARRIVE guidelines recommended for reporting animal research, and Sex and Gender in Research

Laboratory animals	The Cre reporter mice C57BL/6N-Gt(ROSA)26Sortm1(CAG-GCaMP6f)Khakh/J mice is referred to as Gcamp6fl/fl. A transgenic mouse line in which the transgene is under the control of the 3-kb fragment of the human tissue plasminogen activator (Ht-PA) promoter Tg(PLATcre) 116Sdu16 is referred to as Ht-PA::Cre. GCamp6fl/fl males were crossed with Ht-PA::Cre females to generate embryos carrying the calcium fluorescent reporter in neural crest cells and their derivatives. This model was previously described [27]. Heterozygous ls/+ animals were crossed and embryos retrieved at E11.5.
Wild animals	N/A
Reporting on sex	No sex/gender bias (embryo sex was not determined / considered).
Field-collected samples	N/A
Ethics oversight	The methods used to kill the mice conform to the guidelines of CNRS and INSERM animal welfare committees. Killing of mice for retrieval of embryos is a terminal procedure for which neither CNRS or INSERM assign ethics approval codes hence none are given here.

Note that full information on the approval of the study protocol must also be provided in the manuscript.

Plants

Seed stocks	*Report on the source of all seed stocks or other plant material used. If applicable, state the seed stock centre and catalogue number. If plant specimens were collected from the field, describe the collection location, date and sampling procedures.*
Novel plant genotypes	*Describe the methods by which all novel plant genotypes were produced. This includes those generated by transgenic approaches, gene editing, chemical/radiation-based mutagenesis and hybridization. For transgenic lines, describe the transformation method, the number of independent lines analyzed and the generation upon which experiments were performed. For gene-edited lines, describe the editor used, the endogenous sequence targeted for editing, the targeting guide RNA sequence (if applicable) and how the editor was applied.*
Authentication	*Describe any authentication procedures for each seed stock used or novel genotype generated. Describe any experiments used to assess the effect of a mutation and, where applicable, how potential secondary effects (e.g. second site T-DNA insertions, mosiacism, off-target gene editing) were examined.*
